# Effectiveness and Safety of Therapeutic Vaccines for Precancerous Cervical Lesions: A Systematic Review and Meta-Analysis

**DOI:** 10.3389/fonc.2022.918331

**Published:** 2022-06-06

**Authors:** Shan Cai, Xiaoyu Tan, Ke Miao, Dantong Li, Si Cheng, Pei Li, Xueyang Zeng, Feng Sun

**Affiliations:** ^1^ Institute of Child and Adolescent Health, School of Public Health, Peking University, Beijing, China; ^2^ Department of Epidemiology and Biostatistics, School of Public Health, Peking University, Beijing, China; ^3^ Department of Maternal and Child Health, School of Public Health, Peking University, Beijing, China

**Keywords:** precancerous cervical lesions, human papillomavirus, therapeutic vaccines, effectiveness, safety

## Abstract

**Objective:**

This study systematically evaluated the effectiveness and safety of therapeutic vaccines for precancerous cervical lesions, providing evidence for future research.

**Methods:**

We systematically searched the literature in 10 databases from inception to February 18, 2021. Studies on the effectiveness and safety of therapeutic vaccines for precancerous cervical lesions were included. Then, we calculated the overall incidence rates of four outcomes, for which we used the risk ratio (RR) and 95% confidence interval (95% CI) to describe the effects of high-grade squamous intraepithelial lesions (HSILs) on recurrence.

**Results:**

A total of 39 studies were included, all reported in English, published from 1989 to 2021 in 16 countries. The studies covered 22,865 women aged 15–65 years, with a total of 5,794 vaccinated, and 21 vaccines were divided into six types. Meta-analysis showed that the overall incidence rate of HSIL regression in vaccine therapies was 62.48% [95% CI (42.80, 80.41)], with the highest rate being 72.32% for viral vector vaccines [95% CI (29.33, 99.51)]. Similarly, the overall incidence rates of HPV and HPV16/18 clearance by vaccines were 48.59% [95% CI (32.68, 64.64)] and 47.37% [95% CI (38.00, 56.81)], respectively, with the highest rates being 68.18% [95% CI (45.13, 86.14)] for bacterial vector vaccines and 55.14% [95% CI (42.31, 67.66)] for DNA-based vaccines. In addition, a comprehensive analysis indicated that virus-like particle vaccines after conization reduced the risk of HSIL recurrence with statistical significance compared to conization alone [RR = 0.46; 95% CI (0.29, 0.74)]. Regarding safety, only four studies reported a few severe adverse events, indicating that vaccines for precancerous cervical lesions are generally safe.

**Conclusion:**

Virus-like particle vaccines as an adjuvant immunotherapy for conization can significantly reduce the risk of HSIL recurrence. Most therapeutic vaccines have direct therapeutic effects on precancerous lesions, and the effectiveness in HSIL regression, clearance of HPV, and clearance of HPV16/18 is great with good safety. That is, therapeutic vaccines have good development potential and are worthy of further research.

**Systematic Review Registration:**

PROSPERO https://www.crd.york.ac.uk/PROSPERO/, CRD42021275452.

## 1 Background

Cervical cancer is a commonly diagnosed cancer of the reproductive system in women. According to the World Health Organization (WHO), in 2020, 604,127 women were newly diagnosed with cervical cancer and 341,831 women died from the disease throughout the world; the disease ranks fourth in incidence and mortality among all female malignant tumors (1). Cervical cancer has become an important public health problem worldwide. In May 2018, the WHO issued a call to action for the elimination of cervical cancer, and in November 2020, the organization launched a global strategy to accelerate its elimination (2).

Precancerous lesions of cervical cancer refer to the obvious lesions of epithelial cells in the cervical transformation area, of which cytological and histological classifications include cervical intraepithelial neoplasia (CIN), atypical squamous cells of unknown significance (ASCUS), high-grade squamous intraepithelial lesions (HSILs), atypical squamous cells–cannot rule out HSIL (ASC-H), low-grade squamous intraepithelial lesions (LSILs), and atypical glandular cells (AGCs), among others (3). CIN1 is also classified as LSIL, and CIN2–3 are classified as HSIL (3). They are mostly caused by long-term or persistent infection of high-risk types of human papillomavirus (HPV) (3). Because precancerous lesions evolve for years before advancing to invasive cervical cancer, early treatments of precancerous lesions are crucial for preventing disease progression, lowering medical expenditures, and reducing the burden of the disease (4, 5). The WHO recommends cryotherapy, large loop excision of the transformation zone [LLETZ or loop electrosurgical excision procedure (LEEP) in the USA], and cold knife conization as treatments (6). However, complications such as intraoperative and postoperative bleeding, incision infection, cervical stenosis, endometriosis, and intestinal injury may occur (7). There are also higher risks of disease recurrence (8, 9).

As emerging treatment measures for precancerous cervical lesions, therapeutic vaccines have made a lot of progress in recent years. On the one hand, some HPV vaccines have already been used in patients for surgical adjuvant immunotherapy to prevent the recurrence of precancerous lesions. Studies have shown that three HPV vaccines already on the market (Cervarix, Gardasil, and Gardasil 9) can significantly reduce the risk of disease recurrence (10, 11), and it is worth noting that these three vaccines have no direct therapeutic effects. On the other hand, unlike these HPV vaccines, which are designed to induce the production of consistently high levels of neutralizing antibodies, there are therapeutic vaccines that can stimulate cell-mediated immune responses to eliminate HPV-infected cells to directly treat precancerous lesions (12). Several kinds of therapeutic vaccine for precancerous cervical lesions have already been developed, including viral vector, bacterial vector, DNA-based, peptide-based, and protein-based vaccines (13–18), but no systematic review or meta-analysis of related content has been conducted. A comprehensive meta-analysis is urgently needed to evaluate the effectiveness of various therapeutic vaccines for precancerous cervical lesions. Therefore, in this study, we systematically evaluated the effectiveness and safety of therapeutic vaccines for cervical cancer precancerous lesions, establishing a reliable evidence-based basis for the actual effects of therapeutic vaccines, and providing reference for future research.

## 2 Materials and Methods

### 2.1 Search Strategy

We systematically searched the literature in the PubMed, Embase, Web of Science, Cochrane Library, Proquest, ClinicalTrails.gov, Chinese Biomedical Literature Service System (SinoMed), China National Knowledge Infrastructure (CNKI), Chinese Science and Technology Periodicals (VIP), and WanFang databases from inception to February 18, 2021. We combined three key terms in our search strategy, including their subject headings and synonyms: “precancerous cervical lesions,” “vaccine,” and “therapy” (see detailed search strategy in **Supplementary Table S1**). Our results are reported according to the Preferred Reporting Items for Systematic Reviews and Meta-Analyses (PRISMA, see detailed information in **Supplementary Table S2**), and this study has been registered with PROSPERO (CRD42021275452).

### 2.2 Inclusion and Exclusion Criteria

We included published literature related to the effectiveness and safety of therapeutic vaccines in patients with precancerous cervical lesions. Randomized controlled trials (RCTs), non-RCTs, cohort studies, case–control studies, and case series were included. We excluded studies that were not original research, those from which we could not extract outcome data specifically, and those for which we were unable to access the full text. We also excluded mechanism studies, such as animal experiments and *in vitro* experiments, among others.

### 2.3 Literature Screening and Data Extraction

Literature screening and data extraction were conducted in a two-person, independent and parallel manner. Five investigators (ShC, XT, KM, SiC, and DL) screened all identified records independently by reading titles and abstracts. If the information in the title and abstract was insufficient, the full text was obtained for review. If there was any disagreement, it was resolved through discussion with a third person.

The same investigators, working in pairs, read the full texts to extract information independently using a predesigned extraction sheet. The extracted information included basic information (first author and publication year), study design (study type and clinical trial stage), vaccine information (types and name), participants (sample size, age, and disease status), effectiveness and safety outcomes (HSIL regression, HPV clearance, HPV16/18 clearance, HSIL recurrence, and adverse events), and corresponding number of participants.

HSIL regression was defined as histopathological regression to either LSIL or normal pathology (15); HPV clearance was defined as viral clearance based on related HPV genotypes (15); HSIL recurrence referred to new rather than residual HSIL that appeared after treatment (19); any adverse events mainly included any grade 1–4 adverse events; and severe adverse events mainly referred to grade 3–4 adverse events. Grade 1–4 adverse events were classified according to the Medical Dictionary for Regulatory Activities (MedDRA) (20) or the National Cancer Institute Common Terminology Criteria for Adverse Events (CTCAE) (13).

### 2.4 Risk of Bias Assessment

The same five investigators, working in pairs, independently assessed the quality of studies to ensure the reliability of the findings, with disagreements resolved by a third investigator. The Risk of Bias Tool recommended by Cochrane Reviewer’s Handbook (21) was used to assess the quality of RCTs. The Newcastle–Ottawa Scale (NOS) (22) was used to evaluate the quality of cohort studies and case–control studies. For case series studies, we used Recommendations of the National Institute for Clinical Excellence (NICE) (23). For non-RCTs, we used the Bias in Non-randomized Studies - of Interventions (ROBINS) Tool (24).

Cohort studies and case–control studies were classified as having low (scores of 7–9), moderate (5–6), and high risk of bias (0–4) with a total possible score of 9. Case series were classified as having low (6–8), moderate (4–5), and high risk of bias (0–3) with a total possible score of 8.

### 2.5 Statistical Analysis

We calculated the overall incidence rate of effectiveness and safety outcomes. The weight of each study was calculated using the random-effects model directly from the STATA “metaprop” command, and an arcsine transformation was conducted to stabilize variance. Between-study statistical heterogeneity was tested to assess data consistency (the higher the inconsistency, the larger the uncertainty in meta-analysis results) using the *I* ^2^ and Cochran’s *Q* homogeneity tests.

For HSIL recurrence, we used the risk ratio (RR) and its 95% confidence interval (CI) to describe effects. When *I* ^2^ < 50%, indicating low to moderate heterogeneity, a fixed-effects model was used; when *I* ^2^ > 50%, indicating high heterogeneity, a random-effects model was used.

In addition, we conducted a sensitivity analysis by excluding studies with a high risk of bias. Statistical analysis was performed using STATA 15.1 software.

## 3 Results

### 3.1 Basic Characteristics

**Figure 1:**
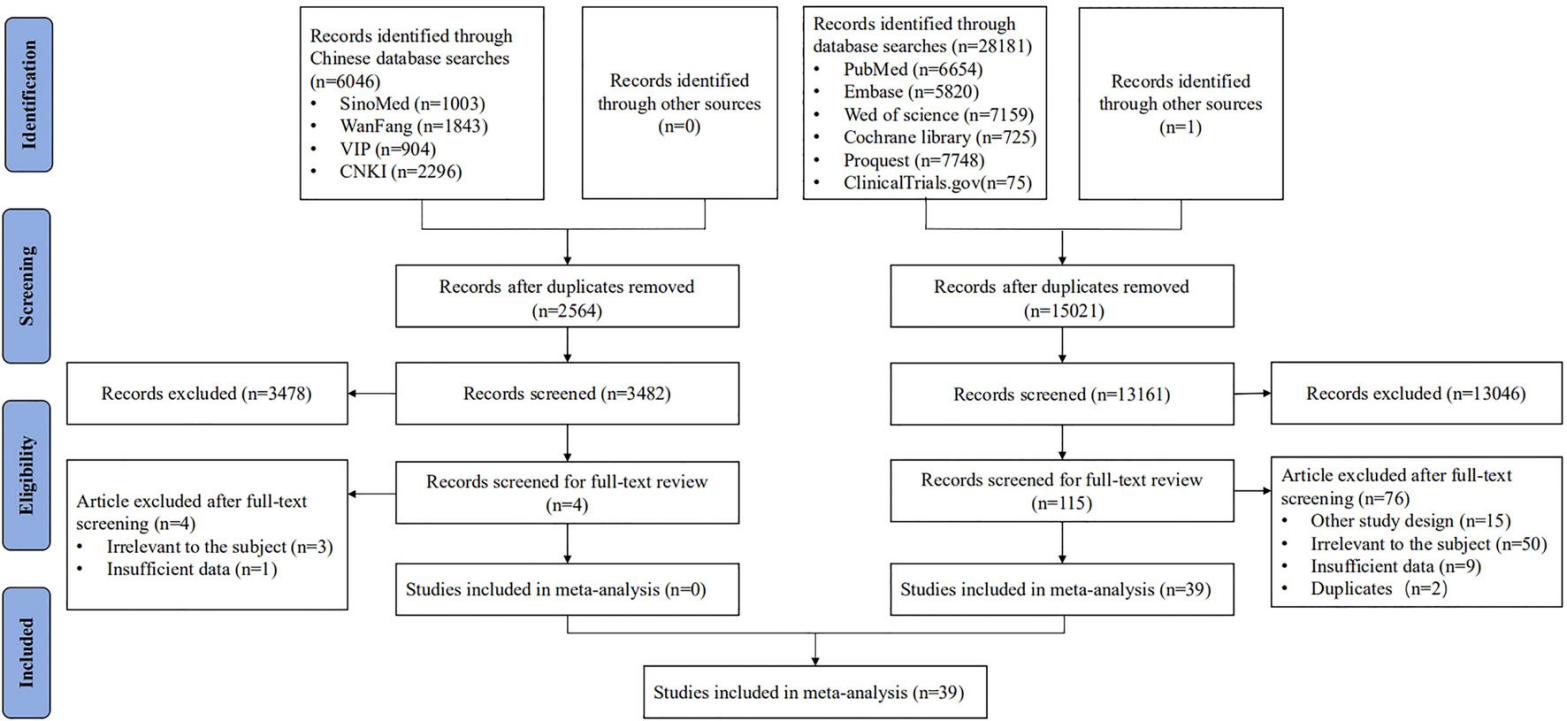
Flowchart of the study selection.

After screening 34,228 articles, 39 studies were included, all reported in English, and published from 1989 to 2021 in 16 countries (**Figure 1**). The study types included RCTs (*n* = 11) (15, 17, 19, 25–32), non-RCTs (*n* = 8) (13, 33–39), case series studies (*n* = 15) (14, 16, 18, 20, 40–50), cohort studies (*n* = 4) (51–54), and case–control studies (*n* = 1) (55). The sample covered 22,865 women aged 15–65, with a total of 5,794 vaccinated. The studies included 21 vaccines divided into six types, including virus-like particle (*n* = 12) (19, 28, 29, 31–34, 51–55), viral vector (*n* = 8) (13, 20, 26, 30, 35, 36, 41, 42), DNA-based (*n* = 7) (15, 27, 37, 40, 41, 43, 46), peptide-based (*n* = 5) (16, 17, 25, 44, 48), protein-based (*n* = 4) (18, 38, 39, 47), and bacterial vector vaccines (*n* = 4) (14, 45, 49, 50). In the included studies, virus-like particle vaccines (Cervarix, Gardasil, and Gardasil 9) were combined with conization to prevent the recurrence of precancerous lesions, whereas other vaccines were used alone as treatment. All studies focused on HSIL as the main disease, and a few studies included other precancerous lesions such as LSIL, ASCUS, ASC-H, and AGC (the basic characteristics of the included studies are shown in **Table 1**).

**Table 1:** Basic characteristics of included studies.

First author (year)	Study type	Clinical trial stage	Age	Sample size	Vaccine type	Vaccine	Diseases	Outcome^a^
Garland (2016) (19)	RCT	Phase III	15–25	T: 190 C: 264	Virus-like particle vaccine	Cervarix	HSIL, LSIL, ASCUS, ASC-H, AGC	(4)
Firnhaber (2020) (28)	RCT	Phase III	/	T: 87 C: 87	Virus-like particle vaccine	Gardasil	HSIL	(4), (5)
Hildesheim (2016) (31)	RCT	Listed	18–25	T: 142 C: 169	Virus-like particle vaccine	Cervarix	HSIL, LSIL	(4)
Karimi-Zarchi (2020) (32)	RCT	Listed	T:22–42 C:22–41	T: 138 C: 104	Virus-like particle vaccine	Gardasil	HSIL, LSIL	(5)
Pieralli (2018) (29)	RCT	Listed	T:23–44 C:23–44	T: 89 C: 89	Virus-like particle vaccine	Gardasil	HSIL, LSIL	(4)
Kang (2013) (33)	Non-RCT	Listed	T:21–45 C:20–45	T: 360 C: 377	Virus-like particle vaccine	Gardasil	HSIL	(4)
Zarochentseva (2020) (34)	Non-RCT	Listed	16–25	T: 100 C: 50	Virus-like particle vaccine	Gardasil	HSIL, LSIL, ASCUS	(6)
Sand (2020) (51)	Cohort study	Listed	T:17–49 C:17–51	T: 2,074 C: 15,054	Virus-like particle vaccine	Unclear^b^	HSIL	(4)
Petrillo (2020) (52)	Cohort study	Listed	T:30–44 C:36–49	T: 182 C: 103	Virus-like particle vaccine	Cervarix, Gardasil	HSIL	(4)
Del Pino (2020) (53)	Cohort study	Listed	/	T: 153 C: 112	Virus-like particle vaccine	Cervarix, Gardasil, Gardasil 9	HSIL, LSIL	(4)
Ortega-Quinonero (2019) (54)	Cohort study	Listed	18–65	T: 103 C: 139	Virus-like particle vaccine	Cervarix, Gardasil	HSIL	(4)
Ghelardi (2018) (55)	Case–control study	Listed	18–45	T: 174 C: 176	Virus-like particle vaccine	Gardasil	HSIL	(4)
Harper (2019) (30)	RCT	Phase IIb	T:18–60 C:19–50	T: 136 C: 70	Viral vector vaccine	Tipapkinogen Sovacivec	HSIL	(1), (5)
Kaufmann (2007) (26)	RCT	Unlisted	/	T: 25 C: 13	Viral vector vaccine	HPV16 L1E7 CVLP	HSIL	(1), (2), (3), (5)
Gutierrez (2004) (35)	Non-RCT	Phase I/II	25–49	T: 36 C: 42	Viral vector vaccine	MVA E2 vaccine	HSIL, LSIL	(1), (2), (3), (5)
Garcia-Hernandez (2006) (36)	Non-RCT	Phase II	25–49	T: 34 C: 20	Viral vector vaccine	MVA E2 vaccine	HSIL	(1), (2), (5)
Rosales (2014) (13)	Non-RCT	Phase III	29–49	T: 1,170 C: 141	Viral vector vaccine	MVA E2 vaccine	HSIL, LSIL	(1), (2), (3), (5)
Komdeur (2021) (42)	Case series	Phase I	25–57	12	Viral vector vaccine	Vvax001	HSIL	(5)
Brun (2011) (20)	Case series	Phase II	25–44	21	Viral vector vaccine	TG4001	HSIL	(1) (2) (3) (5)
Choi (2019) (27)	RCT	Phase II	19–50	1 mg: 36 4 mg: 35^c^	DNA-based vaccine	GX-188E	HSIL	(1) (2) (3) (5)
Trimble (2015) (15)	RCT	Phase IIb	18–55	T: 125 C: 42	DNA-based vaccine	VGX-3100	HSIL	(1) (2) (3) (5)
Alvarez (2016) (37)	Non-RCT	Unlisted	20–44	T1: 11 T2: 11 T3: 10^d^	DNA-based vaccine	pNGVL4a-CRT/E7 (detox)	HSIL	(1) (5)
Bagarazzi (2012) (46)	Case series	Phase I	/	18	DNA-based vaccine	VGX-3100	HSIL	(5)
Trimble (2009) (43)	Case series	Phase I	18–50	15	DNA-based vaccine	pNGVL4a-Sig/E7 (detox)/HSP70	HSIL	(1) (5)
Kim (2014) (40)	Case series	Phase I	23–44	9	DNA-based vaccine	GX-188E	HSIL	(1) (2) (3) (5)
Maldonado (2014) (41)	Case series	Unlisted	22–49	12	DNA-based vaccine +Viral vector vaccine	pNGVL4a-sig/E7 (detox)/HSP70 + TA-HPV	HSIL	(5)
Frazer (2004) (17)	RCT	Phase I	T:19–43 C:23–57	T: 24 C: 7	Peptide-based vaccine	HPV E6 and E7 vaccine	HSIL, LSIL	(5)
de Vos van Steenwijk (2012) (25)	RCT	Phase II	/	T: 5 C: 4	Peptide-based vaccine	HPV E6 and E7 vaccine	HSIL	(5)
Coleman (2016) (44)	Case series	Phase I	22–46	34	Peptide-based vaccine	PepCan	HSIL	(1) (3) (5)
Greenfield (2015) (48)	Case series	Phase I	22–49	24	Peptide-based vaccine	PepCan	HSIL	(1) (2) (5)
Solares (2011) (16)	Case series	Unlisted	24–43	7	Peptide-based vaccine	CIGB-228	HSIL	(1) (2) (3) (5)
Hallez (2004) (38)	Non-RCT	Phase I/II	T:20–42 C:24–35	T: 7 C: 3	Protein-based vaccine	PD-E7/AS02B	HSIL, LSIL	(5)
Simon (2003) (39)	Non-RCT	Unlisted	T:20–51 C:/	T: 5 C: 5	Protein-based vaccine	PD-E7/AS02B	HSIL	(5)
Einstein (2007) (18)	Case series	Phase II	/	58	Protein-based vaccine	SGN-00101	HSIL	(5)
Roman (2007) (47)	Case series	Phase II	/	21	Protein-based vaccine	SGN-00101	HSIL	(1) (2) (5)
Park (2019) (14)	Case series	Phase I/IIa	24–47	19	Bacterial vector vaccine	BLS-M07	HSIL	(1) (5)
Kawana (2014) (45)	Case series	Phase I/IIa	29–43	17	Bacterial vector vaccine	GLBL101c	HSIL	(1) (5)
Klimiek (1989) (50)	Case series	Listed	20–56	35	Bacterial vector vaccine	Gynatren	HSIL, LSIL	(6)
Balajewicz (1989) (49)	Case series	Listed	19–35	30	Bacterial vector vaccine	Gynatren	HSIL, LSIL	(1) (2)

a (1) HSIL regression (2); HPV clearances (3); HPV16/18 clearances (4); HSIL recurrence (5); safety outcomes (6); others.

b One or more of Cervarix, Gardasil, and Gardasil 9 (the text was unclear).

c The study had two intervention groups with different doses of vaccine: 1 mg and 4 mg.

d The study set up three intervention groups using different inoculation modalities: T1: intramuscular injection; T2: cervical intralesional injection; T3: particle-mediated epidermal delivery.

T, vaccinated group; C, non-vaccinated group; RCT, randomized controlled trial; HSIL, high-grade squamous intraepithelial lesion; HPV, human papillomavirus; LSIL, low-grade squamous intraepithelial lesions (LSIL); ASCUS, atypical squamous cells of unknown significance; ASC-H, atypical squamous cells–cannot rule out HSIL; AGC, atypical glandular cells.

In terms of risk of bias, most of the studies had a low/moderate risk. Among the 11 RCTs, 2 studies were judged to be at high risk of bias for incomplete outcome data (25, 32) and 6 studies lacked enough information to judge the risk of bias (17, 19, 26, 27, 30, 31). Among the eight non-RCTs, five studies were judged to have a high risk of bias for unclear measurements or selective reporting of outcomes (13, 35, 36, 38, 39). The bias scores were 4–7 for all 15 case series and 6–7 for the four cohort studies and one case–control study (**Table S3**).

### 3.2 Effectiveness

#### 3.2.1 HSIL Regression

In all, 19 studies reported HSIL regression in 914 patients (13–16, 18, 20, 26, 27, 30, 35–37, 40, 43–45, 47–49). As shown in **Table 2**, a comprehensive analysis showed that the overall incidence rate of HSIL regression induced by the five types of vaccine was 62.48% [95% CI (42.80, 80.41)]. Viral vector vaccines had the best effect, with an incidence rate of 72.32% [95% CI (29.33, 99.51)], followed by protein-based vaccines at rate of 68.91% [95% CI (57.94, 78.96)]. After removing four studies with a high risk of bias, the overall effect changed to 52.08% [95% CI (42.08, 62.01)], and the heterogeneity among studies changed from 96.53% (*p* < 0.01) to 72.66% (*p* < 0.01) (**Table 3**).

**Table 2:** Effectiveness of therapeutic vaccines for precancerous cervical lesions.

Outcome	Vaccine type	No. of studies	n	N	Incidence rate (%) (95% CI)	Weight (%)	*I* ^2^ (%)	*p*-heterogeneity*
HSIL regression								
	Bacterial vector vaccine	3	20	32	66.24 (41.08, 87.86)	14.78	42.01	0.18
	Peptide-based vaccine	3	31	61	50.80 (37.64, 63.90)	15.50	0.00	0.49
	DNA-based vaccine	5	114	227	49.59 (33.34, 65.88)	26.36	76.53	<0.01
	Protein-based vaccine	2	53	78	68.91 (57.94, 78.96)	10.85	0.00	–
	Viral vector vaccine	6	400	516	72.32 (29.33, 99.51)	32.50	98.56	<0.01
	Overall	19	618	914	62.48 (42.80, 80.41)	100.00	96.53	<0.01
HPV clearances								
	Bacterial vector vaccine	1	15	22	68.18 (45.13, 86.14)	7.11	–	–
	Peptide-based vaccine	3	35	60	58.64 (45.30, 71.43)	20.18	0.00	0.62
	DNA-based vaccine	3	98	186	55.14 (42.31, 67.66)	21.57	53.88	0.11
	Protein-based vaccine	2	6	50	10.75 (3.06, 21.48)	14.28	0.00	–
	Viral vector vaccine	5	950	1,219	48.82 (21.83, 76.16)	36.87	94.93	<0.01
	Overall	14	1,104	1,537	48.59 (32.68, 64.64)	100.00	94.51	<0.01
HPV16/18 clearances								
	Peptide-based vaccine	2	6	19	31.11 (11.00, 54.97)	11.72	0.00	–
	DNA-based vaccine	3	98	186	55.14 (42.31, 67.66)	52.49	53.88	0.11
	Viral vector vaccine	4	31	73	41.09 (27.49, 55.23)	35.79	0.00	0.45
	Overall	9	135	278	47.37 (38.00, 56.81)	100.00	32.62	0.16
HSIL recurrence								
	Virus-like particle vaccine	10	T: 159 C: 917	T: 3,552 C: 16,566	T: 4.34 (1.48, 8.46) C: 9.07 (5.42, 13.50)	T: 100.00 C: 100.00	T: 94.27 C: 93.30	T: <0.01 C: <0.01

*The heterogeneity between studies was too low to obtain a p-value because the number of studies was too small (n ≤ 2).

N, total number of participants; n, number of participants with corresponding outcomes; T, vaccinated group; C, nonvaccinated group; HSIL, high-grade squamous intraepithelial lesion; HPV, human papillomavirus.

**Table 3:** Adjusted effectiveness of therapeutic vaccines for precancerous cervical lesions.

Outcome	Vaccine type	No. of studies	n	N	Incidence rate (%) (95% CI)	Weight (%)	*I* ^2^ (%)	*p*-heterogeneity*
HSIL regression								
	Bacterial vector vaccine	3	20	32	66.24 (41.08, 87.86)	15.14	42.01	0.18
	Peptide-based vaccine	3	31	61	50.80 (37.64, 63.90)	19.15	0.00	0.49
	DNA-based vaccine	4	71	156	46.18 (25.08, 67,94)	26.83	76.53	<0.01
	Protein-based vaccine	2	53	78	68.91 (57.94, 78.96)	15.73	0.00	–
	Viral vector vaccine	3	64	167	38.00 (30.55, 45.73)	23.15	0.00	0.43
	Overall	15	239	494	52.08 (42.08, 62.01)	100.00	72.66	<0.01
HPV clearances								
	Bacterial vector vaccine	1	15	22	68.18 (45.13, 86.14)	10.35	–	–
	Peptide-based vaccine	3	35	60	58.64 (45.30, 71.43)	28.78	0.00	0.62
	DNA-based vaccine	2	63	115	55.17 (45.49, 64.67)	20.08	0.00	–
	Protein-based vaccine	2	6	50	10.75 (3.06, 21.48)	20.87	0.00	–
	Viral vector vaccine	2	13	37	35.10 (20.07, 51.64)	19.92	0.00	–
	Overall	10	132	284	45.32 (30.01, 61.06)	100.00	82.45	<0.01
HPV16/18 clearances								
	Peptide-based vaccine	2	6	19	31.11 (11.00, 54.97)	24.41	0.00	–
	DNA-based vaccine	2	63	115	55.17 (45.49, 64.67)	40.33	0.00	–
	Viral vector vaccine	2	13	37	35.10 (20.07, 51.64)	35.26	0.00	–
	Overall	6	82	171	45.41 (31.11, 60.07)	100.00	53.92	0.05

*The heterogeneity between studies was too low to obtain a p-value because the number of studies was too small (n ≤ 2).

N, total number of participants; n, number of participants with corresponding outcomes; HPV, human papillomavirus; HSIL, high-grade squamous intraepithelial lesion.

#### 3.2.2 HPV Clearance

Overall, 14 studies reported HPV clearance in 1,537 patients (13, 15, 16, 18, 20, 26, 27, 35, 36, 40, 44, 47–49), among which 9 reported HPV16/18 clearance in 278 patients (**Table 2**) (13, 15, 16, 20, 26, 27, 35, 40, 44). A comprehensive analysis indicated that the overall incidence rate was 48.59% [95% CI (32.68, 64.64)]. Bacterial vector vaccines had the best effect with a rate of 68.18% [95% CI (45.13, 86.14)], followed by peptide-based vaccines at a rate of 58.64% (95% CI (45.30, 71.43)]. After removing four studies with a high risk of bias, the overall effect changed to 45.32% [95% CI (30.01, 61.06)], and the heterogeneity among studies changed from 94.51% (*p* < 0.01) to 82.45% (*p* < 0.01) (**Table 3**). For HPV16/18 clearance specifically, the overall incidence rate was 47.37% [95% CI (38.00, 56.81)], and DNA-based vaccines had the best effect at a rate of 55.14% [95% CI (42.31, 67.66)]. After removing three studies with a high risk of bias, the overall effect changed to 45.41% [95% CI (31.11, 60.07)], and the heterogeneity among studies changed from 32.62% (*p* = 0.16) to 53.92% (*p* < 0.01) (**Table 3**).

#### 3.2.3 HSIL Recurrence

**Figure 2:**
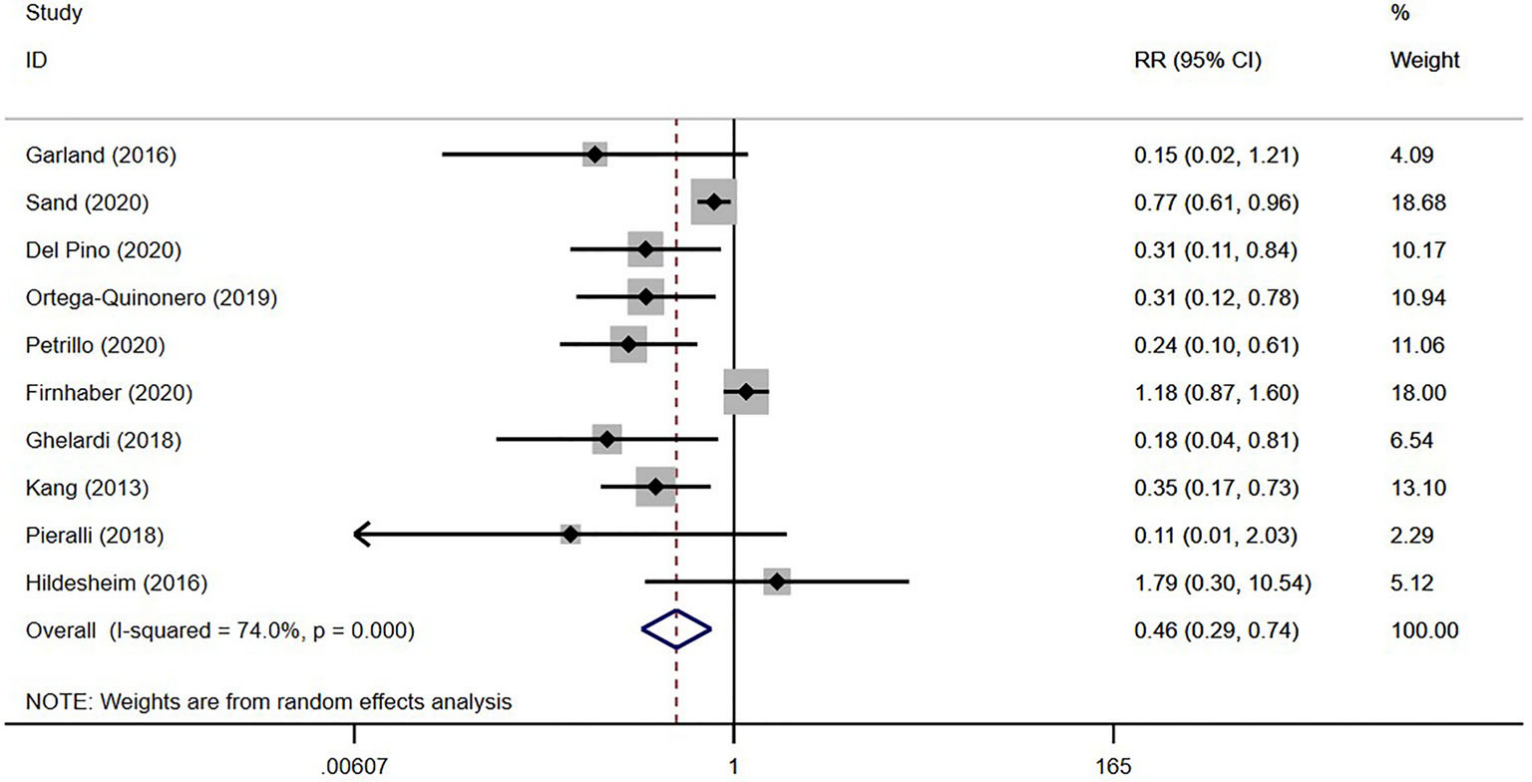
Forest plot for preventing recurrence of HSIL.

In all, 10 studies reported HSIL recurrence in 20,118 patients (19, 28, 29, 31, 33, 51–55), with 3,552 vaccinated. As shown in **Table 2** and **Figure 2**, the virus-like particle vaccines (Cervarix, Gardasil, and Gardasil 9) reduced the risk of recurrent HSIL after conization with an RR of 0.46 [95% CI (0.29, 0.74)], compared to conization alone. All of these 10 studies had low or moderate risk of bias, and the heterogeneity among studies was 74.00% (*p* < 0.01).

### 3.3 Safety

The incidence rates of adverse events after receiving therapeutic vaccines for precancerous lesions of cervical cancer are shown in **Table 4**. The rate of any adverse event was 97.39% [95% CI (92.38, 99.98)]. The rates of any local or systemic adverse events were 85.54% [95% CI (70.16, 96.60)] and 70.42% [95% CI (24.78, 99.98)], respectively, among which injection site pain [89.98%, 95% CI (75.65, 99.04)], injection site redness [55.10%, 95% CI (29.98, 79.03)], and injection site swelling [39.08%, 95% CI (22.18, 57.25)] were common local adverse events and myalgia/muscle pain [40.06%, 95% CI (18.81, 63.30)], fatigue [36.95%, 95% CI (23.45, 51.43)], and headache [31.13%, 95% CI (19.58, 43.86)] were common systemic adverse events. In addition, four studies (15, 17, 25, 30) reported severe adverse events after vaccination: Trimble et al. (15) reported that 2/125 participants treated with VGX-3100 discontinued because of pain; Frazer et al. (17) reported that 1 participant with severe local pain, 1 participant with severe swelling and redness at the injection site, 3 participants with severe tiredness, and 4 participants discontinued because of adverse events after being treated with the HPV E6 and E7 vaccine; de Vos van Steenwijk et al. (25) reported that 2/5 participants discontinued because of adverse events after being treated with the HPV E6 and E7 vaccine; Harper et al. (30) reported a relatively high number of severe adverse events (among 136 patients): 40 with a grade 3 injection site reaction, 1 with lymphadenopathy, and 2 discontinued for adverse events after being treated with the Tipapkinogen Sovacivec vaccine (**Table 5**).

**Table 4:** Safety of therapeutic vaccines for precancerous cervical lesions.

Adverse events	No. of cohorts	n	N	Incidence rate (%) (95% CI)	*I* ^2^ (%)	*p*-heterogeneity
Any adverse events	12	474	496	97.39 (92.38, 99.98)	72.17	<0.01
Any local adverse event	12	266	348	85.54 (70.16, 96.60)	88.27	<0.01
Injection site pain	8	244	271	89.98 (75.65, 99.04)	83.25	<0.01
Injection site redness	7	171	278	55.10 (29.98, 79.03)	92.66	<0.01
Injection site swelling	8	117	280	39.08 (22.18, 57.25)	84.01	<0.01
Any systemic adverse event	3	31	44	70.42 (24.78, 99.98)	86.53	<0.01
Headache	15	162	568	31.13 (19.58, 43.86)	87.33	<0.01
Myalgia/Muscle pain	7	98	284	40.06 (18.81, 63.30)	91.79	<0.01
Flu-like symptoms	10	48	315	19.18 (7.80, 33.45)	83.52	<0.01
Chills	4	22	150	14.79 (0.81, 38.24)	88.78	<0.01
Fever	12	50	345	16.25 (9.20, 24.54)	60.37	<0.01
Fatigue	9	114	248	36.95 (23.45, 51.43)	71.95	<0.01

N, total number of participants; n, number of participants with corresponding outcomes.

**Table 5:** Severe adverse events reported for therapeutic vaccines for precancerous cervical lesions.

First author (year)	Vaccine	Number of people vaccinated	Severe adverse events
Harper (2019) (30)	Tipapkinogen Sovacivec	136	Grade 3 injection site reaction (n = 40); lymphadenopathy (n = 1); discontinued because of adverse events (n = 2).
de Vos van Steenwijk (2012) (25)	HPV E6 and E7 vaccine	5	No second vaccination due to side effects (n = 2).
Frazer (2004) (17)	HPV E6 and E7 vaccine	24	Severe local pain (n = 1); severe swelling and redness at the injection site (n = 1); severe tiredness (n = 3); incomplete series of injections because of adverse events (n = 4).
Trimble (2015) (15)	VGX-3100	125	Discontinued because of pain (n = 2).

## 4 Discussion

To the best of our knowledge, this study is the first meta-analysis on the effectiveness and safety of therapeutic vaccines for precancerous lesions of cervical cancer. We found that therapeutic vaccines for precancerous cervical lesions could be mainly divided into the virus-like particle vaccines listed as adjuvant immunotherapies to prevent recurrence without direct therapeutic effects, and several other unlisted vaccines with direct therapeutic effects on precancerous lesions. Regarding HSIL recurrence, virus-like particle vaccines along with conization significantly reduced the risk of recurrence compared to conization only. In addition, the overall rates of effectiveness of vaccines were 50%–72% (virus vector vaccines were the best) for HSIL regression, 49–68% (bacterial vector vaccines were the best) for HPV clearance except for protein-based vaccines, and 31%–55% (DNA-based vaccines were the best) for HPV16/18 clearance. Safety analysis showed that the incidence rate of adverse events of therapeutic vaccines was high (97.39%), but most of them were mild local adverse events, and severe adverse events were rare.

Studies have reported recurrence rates of 5.3% (56) to 8% (57) for HSIL within 12 months after conization. Therefore, preventing HSIL recurrence is of great significance for improving patient prognosis and quality of life. Prophylactic HPV vaccination after conization is effective for preventing HSIL recurrence (10, 11, 58). A recent meta-analysis showed that the risk of recurrent HSIL was significantly reduced by HPV vaccination [RR = 0.41; 95% CI (0.27, 0.64)], and subgroup analysis showed that it had a more obvious protective effect on HPV16/18 infections [RR = 0.37; 95% CI (0.17, 0.80)], while patient age, vaccination time, and follow-up time had no significant effects on the recurrence rate (10). Our results are similar [RR = 0.46; 95% CI (0.29, 0.74)]. However, due to the inconsistent study designs of the included articles, we could not conduct further analysis of specific vaccines, such as a network meta-analysis to compare the effectiveness of preventing HSIL recurrence among Cervarix, Gardasil, and Gardasil 9.

For the treatment of HSIL, LEEP is currently recommended by the WHO (6). In a previous meta-analysis, the HPV clearance rates of LEEP and cold knife conization for HSIL were 64.71% and 72.27%, respectively. The risks of major bleeding were 0.23% and 0.86%, while the risks of major infections were 0.13% and 0.09%, and the risks of pelvic infectious disease were 0.14% and 0.14%, respectively (56). Another meta-analysis showed that the reporting rate of residual lesions after LEEP was 11.2%, and that after cold knife conization was 6.1% (9). We found that the current therapeutic vaccines for precancerous cervical lesions had an average effectiveness of 62.48% for HSIL regression (the highest was 72.32%), and the average level of HPV clearance was 48.59% (the highest was 68.18%). However, we noted that protein vaccines had great effectiveness in HSIL regression but extremely low pooled effectiveness in HPV clearance. Original articles for calculating pooled effectiveness were two case series studies about SGN-00101 (18, 47). The reason may be that HPV clearance was not clearly associated with HSIL regression or with the immune response evoked by the vaccines, as the virus was not easily removed completely and might be susceptible to reinfection (47). Moreover, another possible reason was that the sample size of the two studies was small and the follow-up time was short (18, 47). In addition, most vaccines are safe, with a high incidence of mild adverse events and a very low incidence of severe adverse events. One article reported 40/136 patients with a grade 3 injection site reaction after being treated with the Tipapkinogen Sovacivec vaccine (30). However, no explanation was given in that study, and the vaccine studied has not yet been marketed. In sum, although the effectiveness of therapeutic vaccines was not as good as that of conization, the gap was not large, and the vaccines showed better safety. That is, therapeutic vaccines have good development potential, and are worthy of further research.

Global HPV screening and preventive HPV vaccination programs have reached great achievements in preventing HPV infection and related diseases (59). However, due to the gap between developed countries and developing countries in terms of screening and access to vaccines, existing HPV vaccines do not eliminate preexisting infections, resulting in a global disease burden for cervical cancer and other related cancers. It is particularly important to continuously explore immunotherapy methods for HPV and related diseases, including therapeutic vaccines. To date, although no specific therapeutic vaccines for precancerous cervical lesions have been marketed, and vaccine treatments have not been included in clinical guidelines (4), numerous clinical trials have shown that some vaccines have great potential for the treatment of precancerous cervical lesions. However, research progress on therapeutic vaccines for precancerous lesions is slow. This may be because the development of vaccines requires more human, material, financial, and time investment, and at the same time faces many difficulties in terms of safety, immunogenicity, HPV polytype, and mutagenicity (60, 61).

There were some limitations to this study that should be mentioned. First, although 39 studies were included, 21 vaccines have been researched in these studies, which means that there is little literature in regard to each vaccine; thus, we only conducted subgroup analysis by vaccine type and risk of bias, and it was difficult to further analyze the age of patients, the severity of precancerous lesions, and the HPV infection status for heterogeneity analysis. Second, because most of the articles that included control groups studied different vaccines and different primary outcome measures, we only calculated the overall incidence rates of most outcomes and did not conduct comparative analyses, including comparison with the HSIL regression that occurs naturally. Previous meta-analysis showed that the pooled rate of lesion regression in untreated CIN2 patients within 24 months was as high as 50%, with high heterogeneity among included studies (62). However, there was little direct evidence for the comparison between therapeutic vaccines and the untreated group; thus, it was difficult to evaluate the effectiveness of therapeutic vaccines in comparison with untreated patients. There was also a lack of comparative studies between different therapeutic vaccines, indicating that high-quality prospective original studies need to be carried out to provide more evidence support for clinical decision-making in the future.

## 5 Conclusion

At present, many studies on therapeutic vaccines for precancerous cervical lesions have been carried out, involving various types of vaccine. Virus-like particle vaccines as an adjuvant immunotherapy for conization can significantly reduce the risk of HSIL recurrence. Most therapeutic vaccines have direct therapeutic effects on precancerous lesions, and the effectiveness in HSIL regression, clearance of HPV, and clearance of HPV16/18 is great. Although the effectiveness of therapeutic vaccines may not be as good as that of conization, the gap is not large, and the vaccines have good safety profiles. That is, therapeutic vaccines have good development potential and are worthy of further research.

## Data Availability Statement

The raw data supporting the conclusions of this article will be made available by the authors, without undue reservation.

## Author Contributions

FS conceived and designed the study. ShC carried out the literature searches. ShC, XT, KM, DL, and SiC extracted the data, and assessed study quality. ShC performed the statistical analysis. ShC, XT, and KM wrote the manuscript. FS, XZ, PL, ShC, XT, and KM revised the manuscript. All authors contributed to the article and approved the submitted version.

## Funding

This study was funded by the National Science and Technology Major Project (2021YFC2301601), the National Natural Science Foundation of China (72074011), and the Special Project for Director, China Center for Evidence Based Traditional Chinese Medicine (2020YJSZX-2). The funders had no role in the study design, data collection or analysis, decision to publish, or preparation of the paper. No payment was received by any of the authors for the preparation of this article.

## Conflict of Interest

The authors declare that the research was conducted in the absence of any commercial or financial relationships that could be construed as a potential conflict of interest.

## Publisher’s Note

All claims expressed in this article are solely those of the authors and do not necessarily represent those of their affiliated organizations, or those of the publisher, the editors and the reviewers. Any product that may be evaluated in this article, or claim that may be made by its manufacturer, is not guaranteed or endorsed by the publisher.
