# Supplementary material for: Effectiveness and Safety of Therapeutic Vaccines for Precancerous Cervical Lesions: A Systematic Review and Meta-Analysis
Source: Front Oncol. 2022 Jun 6;12:918331. doi: 10.3389/fonc.2022.918331 (PMC9207463; doi:10.3389/fonc.2022.918331)
Supplement: Supplementary file 1 [file Table_1.docx]

**Table S1. Search strategies (Pubmed)**

| No. | Query |
| --- | --- |
| 1 | Vaccines[MeSH Terms] |
| 2 | Vaccine[Title/Abstract] |
| 3 | vaccines[Title/Abstract] |
| 4 | #1 OR #2 OR #3 |
| 5 | Uterine Cervical Neoplasms[MeSH Terms] |
| 6 | "Uterine Cervical Neoplasm"[Title/Abstract] |
| 7 | "Uterine Cervical Neoplasms"[Title/Abstract] |
| 8 | "Cervical Neoplasm"[Title/Abstract] |
| 9 | "Cervical Neoplasms"[Title/Abstract] |
| 10 | "Cervix Neoplasm"[Title/Abstract] |
| 11 | "cervical cancers"[Title/Abstract] |
| 12 | "cervical cancer"[Title/Abstract] |
| 13 | "Cervix Neoplasms"[Title/Abstract] |
| 14 | "Cervix Cancer"[Title/Abstract] |
| 15 | "Cervix Cancers"[Title/Abstract] |
| 16 | carcinoma of uterine cervix[Title/Abstract] |
| 17 | cervical intraepithelial neoplasia[MeSH Terms] |
| 18 | cervical intraepithelial neoplasia[Title/Abstract] |
| 19 | Cervical Intraepithelial Neoplasm[Title/Abstract] |
| 20 | carcinoma in situ of cervix[Title/Abstract] |
| 21 | Atypical Squamous Cells of the Cervix[MeSH Terms] |
| 22 | Atypical Squamous Cells of the Cervix[Title/Abstract] |
| 23 | Atypical Cervical Squamous Cell[Title/Abstract] |
| 24 | Atypical Squamous Cell Of Undetermined Significance[Title/Abstract] |
| 25 | Vulvar intraepithelial neoplasia[Title/Abstract] |
| 26 | Vaginal intraepithelial neoplasia[Title/Abstract] |
| 27 | cervical epithelial dysplasia[Title/Abstract] |
| 28 | Uterine Cervical Dysplasia[MeSH Terms] |
| 29 | Uterine Cervical Dysplasia[Title/Abstract] |
| 30 | cervical dysplasia[Title/Abstract] |
| 31 | Squamous Intraepithelial Lesions of the Cervix[MeSH Terms] |
| 32 | Squamous Intraepithelial Lesions of the Cervix[Title/Abstract] |
| 33 | Papillomavirus Infections[MeSH Terms] |
| 34 | "HPV Infection"[Title/Abstract] |
| 35 | "HPV Infections"[Title/Abstract] |
| 36 | #5 OR #6 OR #7 OR #8 OR #9 OR #10 OR #11 OR #12 OR #13 OR #14 OR #15 OR #16 OR #17 OR #18 OR #19 OR #20 OR #21 OR #22 OR #23 OR #24 OR #25 OR #26 OR #27 OR #28 OR #29 OR #30 OR #31 OR #32 OR #33 OR #34 OR #35 |
| 37 | Immunotherapy[MeSH Terms] |
| 38 | Immunotherapy[Title/Abstract] |
| 39 | Immunotherapies[Title/Abstract] |
| 40 | Immunization[MeSH Terms] |
| 41 | Immunization[Title/Abstract] |
| 42 | Immunizations[Title/Abstract] |
| 43 | Vaccination[MeSH Terms] |
| 44 | Vaccination[Title/Abstract] |
| 45 | Vaccinations[Title/Abstract] |
| 46 | Therapeutics[MeSH Terms] |
| 47 | Therapeutic[Title/Abstract] |
| 48 | Therapy[Title/Abstract] |
| 49 | Therapies[Title/Abstract] |
| 50 | Treatment[Title/Abstract] |
| 51 | Treatments[Title/Abstract] |
| 52 | Treat[Title/Abstract] |
| 53 | Prevent[Title/Abstract] |
| 54 | Prevention[Title/Abstract] |
| 55 | Preventions[Title/Abstract] |
| 56 | Preventive[Title/Abstract]\ |
| 57 | #37 OR #38 OR #39 OR #40 OR #41 OR #42 OR #43 OR #44 OR #45 OR #46 OR #47 OR #48 OR #49 OR #50 OR #51 OR #52 OR #53 OR #54 OR #55 OR #56 |
| 58 | #4 AND #36 AND #57 |
