# Supplementary material for: Effectiveness and Safety of Therapeutic Vaccines for Precancerous Cervical Lesions: A Systematic Review and Meta-Analysis
Source: Front Oncol. 2022 Jun 6;12:918331. doi: 10.3389/fonc.2022.918331 (PMC9207463; doi:10.3389/fonc.2022.918331)
Supplement: Supplementary file 3 [file Table_3.docx]

**Table S3. Results of quality assessment**

| Quality assessment of RCTs by the Cochrane Risk of Bias tool | | | | | | | | | | | | | | | | | | | | | | | | | | | | | | | | |
| --- | --- | --- | --- | --- | --- | --- | --- | --- | --- | --- | --- | --- | --- | --- | --- | --- | --- | --- | --- | --- | --- | --- | --- | --- | --- | --- | --- | --- | --- | --- | --- | --- |
| First author (year) | | | | Random sequence generation | | | | Allocation concealment | | | Blinding of outcome assessment | | | | | | Blinding of outcome assessment | | | Incomplete outcome data | | | | | | Selective reporting | | | | Anything else, ideally prespecified | | |
| Choi (2019) | | | | NI | | | | NI | | | NI | | | | | | NI | | | L | | | | | | L | | | | L | | |
| Kaufmann (2007) | | | | NI | | | | NI | | | L | | | | | | L | | | L | | | | | | L | | | | L | | |
| Firnhaber (2020) | | | | L | | | | L | | | L | | | | | | L | | | L | | | | | | L | | | | L | | |
| Harper (2019) | | | | NI | | | | L | | | L | | | | | | L | | | L | | | | | | L | | | | L | | |
| Karimi-Zarchi (2020) | | | | L | | | | L | | | L | | | | | | L | | | H | | | | | | L | | | | L | | |
| de Vos van Steenwijk (2012) | | | | NI | | | | NI | | | NI | | | | | | L | | | H | | | | | | L | | | | L | | |
| Frazer (2004) | | | | NI | | | | L | | | L | | | | | | L | | | L | | | | | | L | | | | L | | |
| Garland (2016) | | | | NI | | | | L | | | L | | | | | | L | | | L | | | | | | L | | | | L | | |
| Hildesheim (2016) | | | | NI | | | | L | | | L | | | | | | L | | | L | | | | | | L | | | | L | | |
| Pieralli (2018) | | | | L | | | | L | | | L | | | | | | L | | | L | | | | | | L | | | | L | | |
| Trimble (2015) | | | | L | | | | L | | | L | | | | | | L | | | L | | | | | | L | | | | L | | |
| Quality assessment of non-RCTs by ROBINS | | | | | | | | | | | | | | | | | | | | | | | | | | | | | | | |  |
| First author | | | Bias due to confounding | | | | Bias in selection of participants into the study | | | | Bias in classification of interventions | | | | | Bias due to deviations from intended interventions | | | | Bias due to missing data | | | | | | Bias in measurement of outcomes | | | Bias in selection of the reported result | | |  |
| Alvarez (2016) | | | L | | | | L | | | | L | | | | | L | | | | M | | | | | | L | | | M | | |  |
| Simon (2003) | | | L | | | | L | | | | L | | | | | L | | | | L | | | | | | H | | | H | | |  |
| Zarochentseva (2020) | | | L | | | | L | | | | M | | | | | L | | | | L | | | | | | L | | | L | | |  |
| Rosales (2014) | | | L | | | | L | | | | L | | | | | L | | | | L | | | | | | H | | | H | | |  |
| Garcia-Hernandez (2006) | | | NI | | | | L | | | | L | | | | | L | | | | NI | | | | | | L | | | H | | |  |
| Gutierrez (2004) | | | NI | | | | L | | | | L | | | | | L | | | | NI | | | | | | L | | | H | | |  |
| Hallez (2004) | | | NI | | | | L | | | | L | | | | | L | | | | NI | | | | | | L | | | H | | |  |
| Kang (2013) | | | L | | | | L | | | | L | | | | | L | | | | L | | | | | | L | | | L | | |  |
| Quality assessment of case series studies by NICE | | | | | | | | | | | | | | | | | | | | | | | | | | | | | | |  |  |
| First author (year) | Case series collected in more than one centre. | | Is the hypothesis/aim/objective of the study clearly described? | | | | | Are the inclusion and exclusion criteria (case definition) clearly reported? | | | | | | Is there a clear definition of the outcomes reported? | | | Were data collected prospectively? | | | | Is there an explicit statement that patients were recruited consecutively? | | | | | Are the main findings of the study clearly described? | | | Are outcomes stratified? | |  |  |
| Komdeur (2021) | 0 | | 1 | | | | | 1 | | | | | | 1 | | | 1 | | | | 0 | | | | | 1 | | | 0 | |  |  |
| Bagarazzi (2012) | 0 | | 1 | | | | | 0 | | | | | | 1 | | | 1 | | | | 0 | | | | | 1 | | | 1 | |  |  |
| Coleman (2016) | 0 | | 1 | | | | | 1 | | | | | | 1 | | | 1 | | | | 0 | | | | | 1 | | | 0 | |  |  |
| Einstein (2007) | 0 | | 1 | | | | | 1 | | | | | | 1 | | | 1 | | | | 0 | | | | | 1 | | | 0 | |  |  |
| Kim (2014) | 0 | | 1 | | | | | 1 | | | | | | 1 | | | 1 | | | | 0 | | | | | 1 | | | 0 | |  |  |
| Brun (2011) | 1 | | 1 | | | | | 1 | | | | | | 1 | | | 1 | | | | 0 | | | | | 1 | | | 0 | |  |  |
| Kawana (2014) | 0 | | 1 | | | | | 1 | | | | | | 1 | | | 1 | | | | 0 | | | | | 1 | | | 0 | |  |  |
| Maldonado (2014) | 0 | | 0 | | | | | 1 | | | | | | 1 | | | 1 | | | | 0 | | | | | 1 | | | 0 | |  |  |
| Park (2019) | 1 | | 1 | | | | | 1 | | | | | | 1 | | | 1 | | | | 0 | | | | | 1 | | | 1 | |  |  |
| Roman (2007) | 1 | | 1 | | | | | 1 | | | | | | 1 | | | 1 | | | | 0 | | | | | 1 | | | 0 | |  |  |
| Solares (2011) | 1 | | 1 | | | | | 1 | | | | | | 1 | | | 1 | | | | 0 | | | | | 1 | | | 1 | |  |  |
| Trimble (2009) | 0 | | 1 | | | | | 1 | | | | | | 1 | | | 1 | | | | 0 | | | | | 1 | | | 1 | |  |  |
| Greenfield (2015) | 0 | | 1 | | | | | 1 | | | | | | 1 | | | 1 | | | | 0 | | | | | 1 | | | 1 | |  |  |
| Balajewicz (1989) | 0 | | 1 | | | | | 1 | | | | | | 0 | | | 1 | | | | 0 | | | | | 1 | | | 1 | |  |  |
| Klimiek (1989) | 0 | | 1 | | | | | 1 | | | | | | 1 | | | 1 | | | | 0 | | | | | 1 | | | 1 | |  |  |
| Quality assessment of cohort studies by NOS | | | | | | | | | | | | | | | | | | | | | | | | | | | | | | | | |
| First author (year) | | | | Representativeness of the exposed cohort | | | Selection of the non-exposed cohort | | | Ascertainment of exposure | | | | Demonstration that outcome of interest was not present at start of study | | | | Comparability of cohorts on the basis of the design or analysis | | | | | | Assessment of outcome | | | Was follow-up long enough for outcomes to occur | | | | | Adequacy of follow up of cohorts |
| Sand (2020) | | | | 1 | | | 1 | | | 1 | | | | 0 | | | | 1 | | | | | | 1 | | | 1 | | | | | 1 |
| Petrillo (2020) | | | | 1 | | | 1 | | | 1 | | | | 0 | | | | 1 | | | | | | 1 | | | 1 | | | | | 1 |
| Ortega-Quinonero (2019) | | | | 1 | | | 1 | | | 1 | | | | 0 | | | | 0 | | | | | | 1 | | | 1 | | | | | 1 |
| Del Pino (2020) | | | | 1 | | | 1 | | | 1 | | | | 0 | | | | 1 | | | | | | 1 | | | 1 | | | | | 1 |
| Quality assessment of case-control studies by NOS | | | | | | | | | | | | | | | | | | | | | | | | | | | | | | | |  |
| First author (year) | | | Is the Case Definition Adequate? | | Representativeness of the Cases | | | Selection of Controls | | | | Definition of Controls | | Comparability of Cases and Controls on the Basis of the Design or Analysis | | | | | | | Ascertainment of Exposure | | | Same method of ascertainment for cases and controls | | | | Non-Response Rate | | | |  |
| Ghelardi (2018) | | | 1 | | 0 | | | 0 | | | | 1 | | 2 | | | | | | | 1 | | | 1 | | | | 1 | | | |  |
